# Closer to personalized approach through accepting the gender differences in medicine

**DOI:** 10.1186/1878-5085-5-S1-A148

**Published:** 2014-02-11

**Authors:** Hele Everaus

**Affiliations:** 1 University of Tartu, Tartu University Hospital, Estonia

## 

Personalized medicine is rapidly advancing field of healthcare. Each person is unique by the genetic, genomic, proteomic and metabolomics background, which are playing important role also in the individual disease mechanisms and the efficacy of the treatment. Besides all abovementioned aspects the impact of gender on the pathogenetic mechanisms of different diseases is not often considered.

## The aim of the study

Immune status of the individual is playing important role in health and disease. The differences in immune responses between males and females have been reported. It has been proposed that sex hormones and their balance may influence the immune system.

The aim of study was to examine possible gender differences in one of the malignancies – chronic lymphocytic leukemia (CLL). The status of the immune – hypothalamic-pituitary-gonadal circuit and immune activity was investigated.

## Material and methods

132 patients with CLL, 76 males and 56 females were studied. 38 healthy age and sex matched subjects served as controls. Immune status was investigated by mitogen stimulation of peripheral blood lymphocytes. Phytohemagglutinin (PHA), concanavalin A (Con A) dextran sulphate (DxS) were used as mitogens. An optimized whole blood method to study the mitogenic lymphocytic proliferation was used [1]. Proliferation was measured by 3H-thymidine uptake and assessed autoradiographically. Results were expressed in mean stimulation index. To study the hormonal balance concentrations of plasma adrenocorticotropic hormone (AKTH), serum cortisol (C), follicle stimulating hormones (FSH), luteinizing hormone (LH), testosterone (T) and 17ß-estradiol were measured.

## Results

CLL patients demonstrated significant decrease of T-lymphocytes function. Remarkable increase of serum cortisol level was found in CLL patients, whereas no changes in ACTH concentrations were demonstrated. We can guess the existence of serious dysregulation of the hypothalamic-pituitary-adrenal axis. Values of FSH, LH and E in men with CLL were significantly higher than in healthy persons. No significant differences were demonstrated in the levels of abovementioned hormones in female CLL patients. The dysbalance of sex hormones can influence the immune functions. Negative correlation has been demonstrated between E/T radio and T-lymphocyte function in male CLL patients. We can guess that earlier observed worse survival of male CLL patients compared to females can be connected with the impact of sex hormones dysbalance on immune regulation and through that to overall pathogenesis of disease. Males and females normally have dimorphic immune response. It is reasonable to suggest that in pathologic conditions (for example in CLL) immune regulation is altered in different ways in males and females. Sex hormones dysbalance in CLL can be responsible for that. Further studies are needed to clarify whether CLL male and female patients would need different therapy in connection with diversity of pathogenesis and disease progression.

In conclusion, there are some arguments presented that gender-based medicine will become irrelevant if all individual factors can be taken into account. However, large databases reveal that gender remains as an independent risk factor. It is important to consider and find out the impact of gender differences in individualised medicine approach.
